# Preserved Error-Monitoring in Borderline Personality Disorder Patients with and without Non-Suicidal Self-Injury Behaviors

**DOI:** 10.1371/journal.pone.0143994

**Published:** 2015-12-04

**Authors:** Daniel Vega, Adrià Vilà-Balló, Àngel Soto, Julià Amengual, Joan Ribas, Rafael Torrubia, Antoni Rodríguez-Fornells, Josep Marco-Pallarés

**Affiliations:** 1 Department of Psychiatry and Mental Health, Consorci Sanitari de l'Anoia, Igualada, Barcelona, Spain; 2 Unitat de Psicologia Mèdica, Departament de Psiquiatria i Medicina Legal & Institut de Neurociències, Universitat Autònoma de Barcelona, Bellaterra, Barcelona, Spain; 3 Cognition and Brain Plasticity Group, Bellvitge Biomedical Research Institute- IDIBELL, L’Hospitalet de Llobregat, Barcelona, Spain; 4 Department of Basic Psychology-Campus Bellvitge, University of Barcelona, L’Hospitalet de Llobregat, Barcelona, Spain; 5 Catalan Institution for Research and Advanced Studies (ICREA), Barcelona, Spain; 6 Institut de Recerca en Cervell, Cognició i Conducta, IR3C, University of Barcelona, Barcelona, Spain; Central Institute of Mental Health, GERMANY

## Abstract

**Background:**

The presence of non-suicidal self-injury acts in Borderline Personality Disorder (BPD) is very prevalent. These behaviors are a public health concern and have become a poorly understood phenomenon in the community. It has been proposed that the commission of non-suicidal self-injury might be related to a failure in the brain network regulating executive functions. Previous studies have shown that BPD patients present an impairment in their capacity to monitor actions and conflicts associated with the performance of certain actions, which suppose an important aspect of cognitive control.

**Method:**

We used Event Related Potentials to examine the behavioral and electrophysiological indexes associated with the error monitoring in two BPD outpatients groups (17 patients each) differentiated according to the presence or absence of non-suicidal self-injury behaviors. We also examined 17 age- and intelligence- matched healthy control participants.

**Results:**

The three groups did not show significant differences in event-related potentials associated with errors (Error-Related Negativity and Pe) nor in theta power increase following errors.

**Conclusions:**

This is the first study investigating the behavioral and electrophysiological error monitoring indexes in BPD patients characterized by their history of non-suicidal self-injury behaviors. Our results show that error monitoring is preserved in BPD patients and suggest that non-suicidal self-injury acts are not related to a dysfunction in the cognitive control mechanisms.

## Introduction

Borderline Personality Disorder (BPD) is the most common personality disorder, affecting about 0.5 to 5.9% of the general population [1]. One of the most characteristic and common symptoms in BPD is the presence of non-suicidal self-injury (NSSI) behaviors [2], which refers to the deliberate, self-inflicted destruction of body tissue without suicidal intent, and for purposes not socially sanctioned (*e*.*g*. tattoos or piercings) [3,4]. Because NSSI behaviors are a public health concern [5], they have become a new clinical entity in the new DSM-5 [6], in contrast to DSM-IV-TR [7], in which they were only restricted to the BPD. Despite growing scientific interest, little is known about the reason why people engage in a direct form of self-injury against the innate fight for self-preservation [8].

BPD patients usually carry out NSSI behaviors during states of emotional stress as a maladaptive attempt to self-regulate [9–11]. It has been proposed that these behaviors might be explained by a failure in the executive functioning involved in emotion regulation and cognitive control [12,13]. Dysfunction in executive processing might be at the core of some of the BPD symptoms, especially impulsivity and emotion regulation among others [14], and it has also been related with NSSI beyond the BPD [15].

One of the most important subcomponents of cognitive control is the capacity to monitor errors and conflicts associated with the performance of certain actions (also referred to as ‘response monitoring’ or ‘performance monitoring’) [16]. A well-known electrophysiological signature of these functions is the Error-Related Negativity (ERN, also known as Ne) [17,18], an Event-Related Potential (ERP) which appears after the execution of an error in a speeded-up action-selection task. The ERN peaks 60–80 ms after the erroneous response and shows a frontocentral scalp distribution consistent with a neural source in the Anterior Cingulate Cortex (ACC) [19]. While first accounts interpreted this component as an error execution index [18], recent theories have related it to different functions such as conflict detection [20] or reinforcement-learning teaching signals indexing worse than expected events [21]. In addition, another ERP component, the so-called error positivity Pe, appears around 300 ms after the execution of an error [22]. This ERP component shows a centro-parietal topography and has been related to error awareness [23,24].

Error-processing dysfunctions have been reported in a variety of mental disorders when compared with healthy controls (for a review: [25] and [26]). In BPD, this alteration is manifested by an increase in the reaction time (RT) of erroneous responses compared to correct ones and attenuated ERN [27], but not Pe amplitude [28]. In these patients reduced ERN amplitude has been related to self-reported impulsivity [28]. Surprisingly, no previous studies have investigated ERP error monitoring signatures associated with NSSI behaviors despite their relationship with executive functions. Thus, NSSI acts are impulsive [29,30] and repetitive maladaptive coping responses to stressful situations [4], which suppose a non-optimal response to outcomes [31]. Due to overlapping between NSSI and BPD (69–90% of BPD patients engaged in NSSI) [32], it is difficult to establish to what extent the impairment in error monitoring found in previous BPD studies [27,28] is specific to this disorder or, in contrast, is related to NSSI.

The goal of the present study was to determine the impairment of error monitoring and cognitive control in BPD patients according to their tendency to engage in NSSI behaviors. Following previous studies we hypothesized that BPD patients (when compared to healthy controls) would present a reduced ERN after error execution indicating impairment in cognitive control [27,28]. In addition, we hypothesized those BPD patients with NSSI history would show a larger reduction in ERN and Pe components compared to those without it, thus indicating a more severe impairment in the cognitive control system.

## Materials and Methods

### Participants

All procedures were approved by the Clinical Research Ethics Committee of the Bellvitge University Hospital and the study was conducted in accordance with the Declaration of Helsinki. An information sheet about the study was given to all patients and healthy controls. Participants were encouraged to discuss their possible participation with close people and take several days to decide it. Obtaining informed consent involved the following steps. First, a member of the research staff discussed the study with each of the potential participants ensuring that they understood the procedures, risks, and benefits as well as the fact that their participation was voluntary and their refusal would have no consequences. In this interview, participants were encouraged to ask any questions they might have. Based on this interview, only the subjects who had the mental faculties to consider their participation and their decision-making capacity intact were recruited to the study. Finally, written informed consent was obtained from all subjects who agreed to participate. Each participant freely signed the informed consent form.

Two groups of 17 BPD outpatients each were selected. All patients were women, and they were in treatment in the Mental Health Area of the Hospital of Igualada (Spain). Table 1 shows the demographical and clinical characteristics of these groups. The Diagnostic Interview for Borderlines-Revised (DIB-R) [33] was used twice with two independent trained clinicians each, in order to ensure the diagnosis (first: 7.85±1.21; second: 7.82±1.26; Intraclass Correlation Coefficient = .58). Both groups were created according to the presence or not of NSSI. Thus, we selected a BPD group (SI-BPD; N = 17) characterized by: a) lifetime history of five or more episodes of any NSSI behavior (determined by the Inventory of Statements About Self-injury, ISAS, see below), and b) two of these episodes occurred in the last two years (determined by the self-harm item of the DIB-R). In contrast, the BPD group without NSSI (NI-BPD; N = 17) was composed of BPD patients with no prior history of any NSSI behavior at the time of study enrollment (assessed by the ISAS and DIB-R). The NSSI typologies and frequency depicted in Table 2. In addition, both groups were matched in sex, age and IQ (Table 1). Finally, a third group of seventeen sex-, age-, and IQ-matched control women, were recruited by means of local advertising. These participants had no previous history or current mental disorder.

**Table 1:** Demographic and Clinical Characteristics of BPD patients and Healthy control participants.

	NI-BPD (n = 17)		SI-BPD (n = 17)		Controls (n = 17)		Group differences	
	Mean	SD	Mean	SD	Mean	SD	*F*	*p*
**Participants Characteristics**								
Age (years)	30.29	6.26	29.94	6.04	33.18	6.38	1.38	.261
IQ	101.08	10.06	94.96	8.19	99.52	8.68	2.12	.131
Onset^a^ (age)	27.06	5.01	25.47	5.21			.79	.378
**Clinical status**								
BIS-11	69	17.52	76.25	17.28	41.23	15.11	20.46	**< .001** ^**b**^
HDRS	10.06	5.87	13.06	3.68			3.13	.087
GAF	56.04	7.98	47.96	6.84			3.81	**< .001**
DIB-R	7.37	1.02	8.29	1.05			6.48	**.016**
CGI-BPD	4.51	1.41	5.65	0.99			7.32	**.011**
BSL-23	2.01	0.83	2.11	0.97			.33	.73

Notes. *BIS-11*, Barratt Impulsiveness Scale-11; *HDRS*, Hamilton Depression Rating Scales; *GAF*, Global Assessment of Functioning (DSM-IV); *DIB-R*, Diagnostic Interview for Borderlines-Revised; *CGI-BPD*, Clinical Global Impression for the BPD; *BSL-23*, Borderline Symptom List 23.

^a^ Age at onset of any regular BPD treatment

^b^ SI-BPD = NI-NSSI, BPD > Control.

Significant values were depicted in bold. *P*-values lower than .001 are indicated as < .001.

**Table 2:** Lifetime frequency of 12 NSSI behaviors assessed by the ISAS.

	NSSI Behaviors											
	cutting	burning	scratching	banging	biting	carving	wound picking	needle-sticking	pinching	hair pulling	Rubbing ^a^	Chemicals ^b^
	N (%)	N (%)	N (%)	N (%)	N (%)	N (%)	N (%)	N (%)	N (%)	N (%)	N (%)	N (%)
<5	3 (17.6)	5 (29.4)	15 (88.2)	11 (64.7)	12 (70.6)	10 (58.8)	12 (70.6)	2 (11.8)	7 (41.2)	10 (58.8)	16 (94.1)	15 (88.2)
5–50	6 (35.3)	7 (41.2)	1 (5.9)	4 (23.5)	3 (17.6)	3 (17.6)	3 (17.6)	9 (52.9)	4 (23.5)	3 (17.6)	1 (5.9)	1 (5.9)
51–100	1 (5.9)	2 (11.8)	0	0	0	2 (11.8)	2 (11.8)	2 (11.8)	2 (11.8)	1 (5.9)	0	0
101–250	2 (11.8)	1 (5.9)	1 (5.9)	0	0	0	0	1 (5.9)	2 (11.8)	2 (11.8)	0	0
>250	5 (29.4)	2 (11.8)	0	2 (11.8)	2 (11.8)	2 (11.8)	0	3 (17.6)	2 (11.8)	1 (5.9)	0	1 (5.9)
Total > 5	14 (82.4)	12 (70.6)	2 (11.8)	6 (35.3)	5 (29.4)	7 (41.2)	5 (29.4)	15 (88.2)	10 (58.8)	7 (41.2)	1 (5.9)	2 (11.8)

Notes. BPD subjects estimated the number of times they have engaged NSSI behaviors. The total score was grouped in different categories (from less than 5 times to more than 250 times). Additionally the lifetime frequency above 5 for each NSSI type was computed.

^a^ Rubbing skin against rough surfaces

^b^ Swallowing chemicals

All three groups were assessed with the Spanish version of the Structured Clinical Interview for DSM-IV Axis II Personality Disorders [34] and with DSM-IV Axis I interview [35]. BPD patients showed comorbidity with other personality disorders and Axis I disorders (for further information see Table B in S1 File). Finally, the presence of brain injury, psychotic, bipolar, current major depressive disorder or drug abuse and IQ below 80 were exclusion criteria.

### Psychometric measures

First, a Spanish version of the Inventory of statements about self-injury (ISAS) [36] was used to assess the lifetime frequency of 12 NSSI behaviors (*e*.*g*. cutting, burning or carving) and their descriptive and contextual factors (*e*.*g*. age of onset). This part of the ISAS shows good reliability and validity [37]. Those respondents who endorsed one or more NSSI behaviors were instructed to complete the second part of the ISAS, which assesses 13 potential functions of these NSSI behaviors (e.g. sensation seeking, affect regulation). Participants also completed the Borderline Symptom List (BSL-23) [38,39], which evaluates the amount of suffering on a list of 23 problems during the last week (e.g., “It was hard for me to concentrate” or “I wanted to punish myself”). In addition, the CGI-BPD severity form scale, which is an adaptation of the Clinical Global Impression (CGI) scale designed to assess severity in BPD patients [40], was completed by the clinician. Finally, the Barratt Impulsiveness Scale (BIS-11) [41] was used to measure the impulsivity of the patients.

### Medication load

A medication load protocol was used to determine the total medication load, as previously used in psychiatric population [42]. Anti-depressant, anxiolytic, mood stabilizer, and anti-psychotic medications were coded as absent = 0, low = 1, or high = 2 based on previously employed methods to convert each medication to a standardized dose [43,44]. Anti-psychotics were converted into chlorpromazine dose equivalents [45]. As a result, a composite measure of total medication load was obtained.

### Task

We applied a modified variant of the Eriksen Flanker task [46] that required the participants to respond, pressing the corresponding mouse button with the index or middle finger of their dominant hand, to the pointing direction (right or left) of a central arrow from an array of five arrows. All four surrounding arrows were either compatible or incompatible with the central arrow (same or different direction respectively), favoring performance errors in the incompatible condition [47,48]. We presented 33.3% of compatible and 50% of incompatible trials. In the remaining 16.6%, we included no-go trials, following a variant of the stop-signal paradigm [49]. In these stop trials, the central green arrow changed to red after a variable delay, indicating that participants should inhibit their response. The delay was adapted to participants’ behavior by means of a staircase tracking algorithm [50] functioning as follows. The stop-signal delay was set to 140 ms initially. After a successful inhibition the stop-signal delay was increased by 10 ms (making the inhibition harder). After an inhibitory failure the stop-signal delay was reduced by 10 ms (making inhibition easier). This procedure was applied to yield an inhibition rate of 50%.

We computed the stop-signal reaction time (SSRT) [49] by subtracting the participant’s mean stop-signal delay from the median reaction time of correct go responses. Each stimulus array was presented in the middle of the screen. Stimulus duration was 300 ms and the stimulus onset asynchrony was fixed to 900 ms. Participants received at least 20 training trials to get acquainted to the task. They were encouraged to correct their errors in the go trials as fast as possible, even though no performance feedback was given to participants. The experiment was divided into 55 blocks, each comprising 20 trials, resulting in a total of 1100 trials. After each block, a rest period of 13 seconds was included.

### Electrophysiological Recording

The electroencephalographic (EEG) activity was recorded continuously (digitized with a sampling rate of 250 Hz, high-pass band at 0.01 Hz, notch filter) using SYNAMP Neuroscan amplifiers from 28 tin electrodes, mounted in an elastic cap and located at standard positions (FP1/2, F3/4, C3/4, P3/4, FCz, T3/4, F7/8, T5/6, Fz, Cz, Pz, FC1/2, FC5/6, CP1/2, CP5/6, PO1/2). The EEG was referenced on-line to the right ocular canthus. Biosignals were re-referenced offline to the mean of the activity at the two mastoid processes and high-pass filtered at 1 Hz. Electrode impedances were kept below 5 kΩ. Vertical eye movements were monitored by an electrode placed below the right eye. Finally, the SOBI algorithm proposed in Joyce et al. [51] was used to automatically remove eye movements and blink artifacts.

### Data analysis

In the behavioral and ERP analyses, exceptionally slow (2.5 SD) and fast (<120 ms) reaction time (RT) responses were not included to the analysis, with no group differences on the percentage of removed trials [Control: 2.57 ± .72%, SI-BPD: 2.31 ± .56%, and NI-BPD: 2.81 ± .81%; *F*(2,48) = 2.087, *p* = .135]. The included behavioral data was decomposed in different variables. Two groups of variables were reported; on one hand the RTs, for correct and error responses, for compatible correct and incompatible correct responses, together with the post-error-slowing and the stop-signal RT. On the other hand, different percentages were reported: the percentage of correct and error trials, taking account all the included behavioral go-trials; the percentage of compatible correct and incompatible correct, taking account the total included in each condition, respectively; the total of inhibited and non-inhibited, taking account all the included behavioral stop-trials; and finally; the percentage of corrected errors on the total of included behavioral errors was reported. Each variable dependent variable was submitted to a one-way ANOVA with Group (Controls, SI-BPD, NI-BPD) as a 3-level factor. Table 3 contains descriptive and statistical information of the behavioral data.

**Table 3:** Flanker Task Behavioral results.

	Controls (N = 17)		SI-BPD (N = 17)		NI-BPD (N = 17)		Group effect	
	M	D	M	SD	M	SD	*F* (2,48)	*P* value
**RT (*ms*.*)***								
Correct	450.73	63.19	483.24	65.63	468.99	50.43	1.249	.296
Error	319.60	39.99	330.47	48.09	361.00	64.42	2.914	.064
Compatible Correct	433.80	60.39	464.46	69.57	456.23	53.88	1.127	.332
Incompatible Correct	462.33	65.77	496.41	64.04	478.16	49.16	1.368	.264
Post-error-slowing	41.03	25.53	23.51	36.09	31.54	34.48	1.248	.296
SSRT	297.09	58.79	328.50	66.98	316.48	50.67	1.219	.305
**Response (%)**								
Total Correct	95.76	4.60	95.48	3.12	94.01	5.83	.695	.504
Total Error	4.25	4.60	4.52	3.12	5.99	5.83	.695	.504
Compatible Correct	97.23	3.34	97.35	2.31	96.88	4.37	.086	.917
Incompatible Correct	94.93	5.69	94.17	4.01	92.03	8.07	1.019	.369
Inhibited	38.17	22.00	37.27	14.58	38.79	15.01	.032	.969
Non-Inhibited	61.80	21.98	62.71	14.55	61.13	15.08	.035	.966
Corrected errors	56.13	36.43	64.37	30.06	57.13	30.29	.328	.722

Notes. Means of Reaction times (RT; for each condition, post-error-slowing and SSRT) and of percentage of Responses, in the performance of the Flanker Task. Data are depict for each group, and can be observer the corresponding ANOVA with associated P values.

ERP averages were also obtained for the different conditions (time-range from -400 to 600 ms for response-locked ERPs), with a baseline of 50 ms before the button press. Epochs exceeding ±100 μV in electrooculogram (EOG) or EEG were removed from further analysis. Importantly, no group differences were found regarding the total of rejected epochs [Control: 5.25 ± 7.51%, SI-BPD: 11.02 ± 15.36%, and NI-BPD: 8.61 ± 12.45%; *F*(2,48) = .957, *p* = .391]. All artifact-free error trials were included regardless of a subsequent corrective response. As previous studies have shown that both choice–errors and stop-errors present similar EEG responses both in normal [52] and in clinical [53] population, we combined the choice-errors and stop-errors together in the ERP analysis in order to increase the number of error trials.

Repeated-measures ANOVAs with Condition (compatible, incompatible), Electrode location (Fz, Cz, Pz) and Response (correct, incorrect), as within-subject factors and Group (Control, SI-BPD and NI-BPD) as between-subject factors were performed using the Greenhouse-Geisser epsilon correction as appropriate [54]. The corrected *P*-value is reported. Finally, to discard possible effects of medication, a Pearson’s correlation analysis was carried out between the Medication Load scale and the amplitude of ERN and Pe components, and also the theta band of Time-Frequency.

Time-Frequency of the electrical activity elicited by the errors and the correct responses were generated (epochs comprising 4000 ms; 2000 ms before and after the response). Epochs exceeding ±100 μV in EOG or EEG were removed from further analysis. Baseline was the 100 ms prior the button press. Single trial data was convoluted using a 7-cycles complex Morlet wavelet [55]. Changes in time varying energy (square of the convolution between wavelet and signal) in the studied frequencies (from 1Hz to 40Hz; linear increase) with respect to baseline were computed for each trial and averaged for each subject before performing a grand average.

## Results

### Psychometric results

The psychometric results are depicted in Table 1. As it shows, the SI-BPD group obtained a higher overall score than the NI-BPD group in the diagnostic interview (DIB-R). Congruently, the severity indices showed higher severity (CGI-BPD) and less functionality (GAF) of SI-BPD than NI-BPD group. Contrarily, both groups did not show statistical differences in current depressive symptoms (HDRS) or in the self-reported measures of clinical state (BSL-23) and impulsivity (BIS-11).

### Behavioral results

Behavioral data details were depicted in Table 3. Participants responded faster to compatible (451.50 ± 61.77 ms) than to incompatible (478.97 ± 60.56 ms) trials [main effect of Condition, *F*(1,48) = 160.622, *p* < .001, *Ƞ* ^2^ = .770]. Importantly, no significant differences were found between groups [Group: *F*(2,48) = 1.235, *p* = .300, *Ƞ* ^2^ = .049; Condition x Group: *F*(2,48) = 1.841, *p* = .170, *η* ^*2*^ = .071; Figure A in S1 File].

No statistical differences were found between groups in the percentages of correct trials and correction after errors (F<1.7). Furthermore, importantly, no SSRT differences were found between groups. In consequence, the three groups were showed very similar in their behavioral performance in the Flanker task (for a visual analyses see Figure A in S1 File).

### Response-locked ERP data

**Fig 1:**
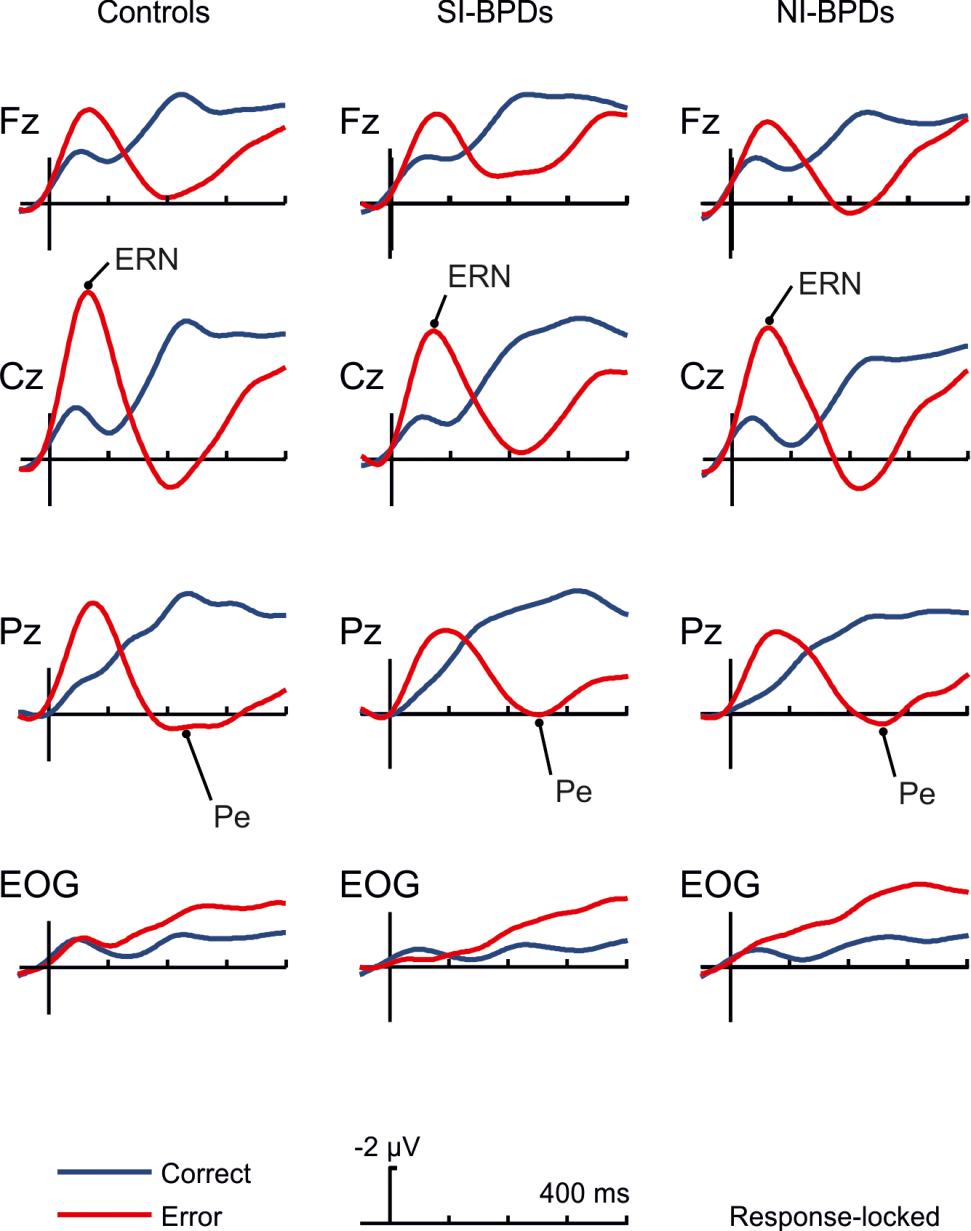
Grand average of response-locked ERPs at Fz, Cz, Pz and EOG electrodes for controls, SI-BPD and NI-BPD individuals. Correct trials are depicted in blue solid lines, and choice/stop-error trials in red lines. Data were low-pass filtered at 12 Hz for illustration purposes.

**Fig 2:**
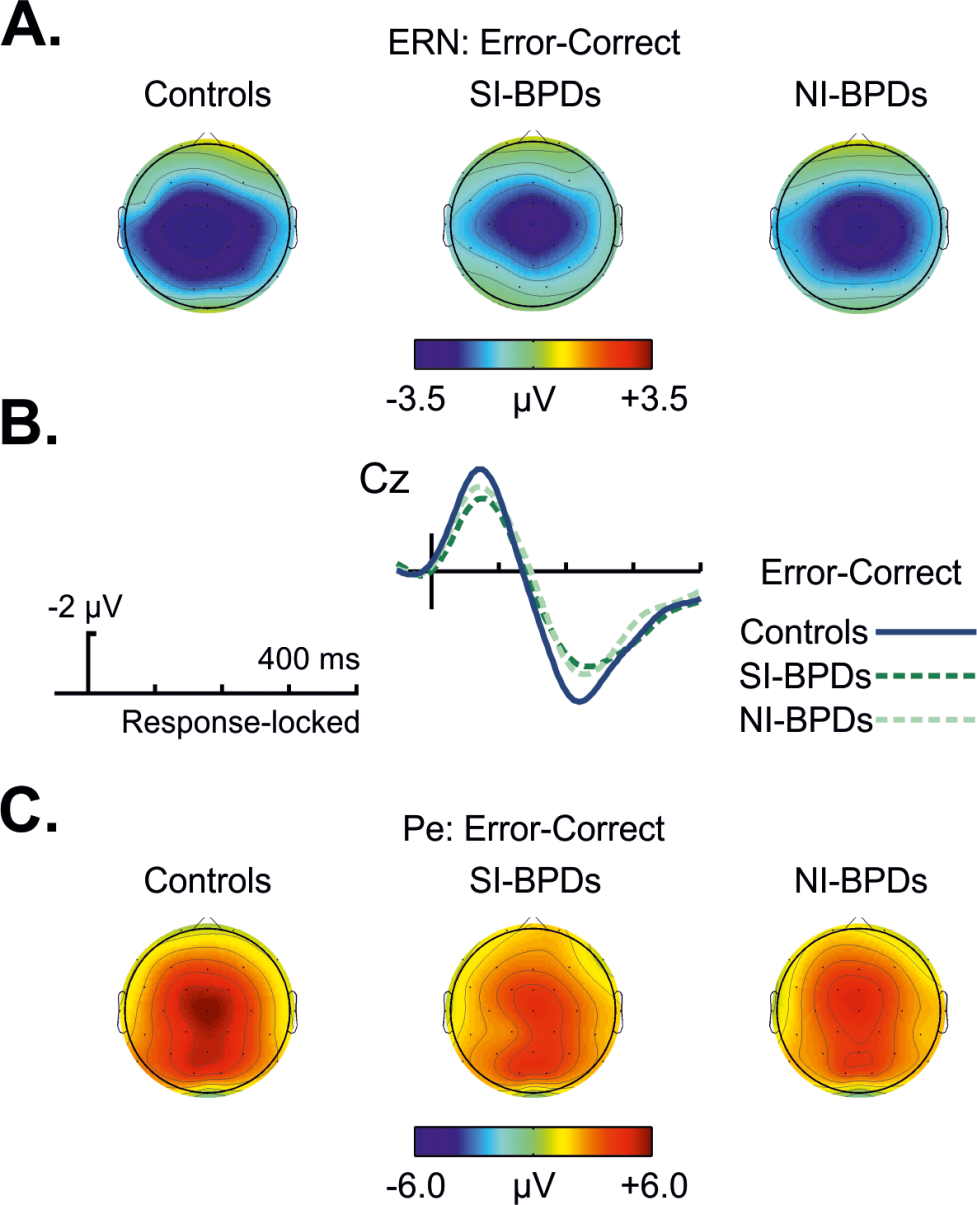
(A) Topography for error *vs* correct for the time window 20–70 ms (maximum and minimum values in microvolts are -3.5 and +3.5). (B) Differences waveform for the grand average between the error and correct trials, at Cz electrode, for controls (blue solid line), SI-BPD (hard green line), and NI-BPD (light green line) individuals. (C) Topography for error *vs* correct for the time window 170–270 ms, maximum and minimum values in microvolts are -6.0 and +6.0.

**Fig 3:**
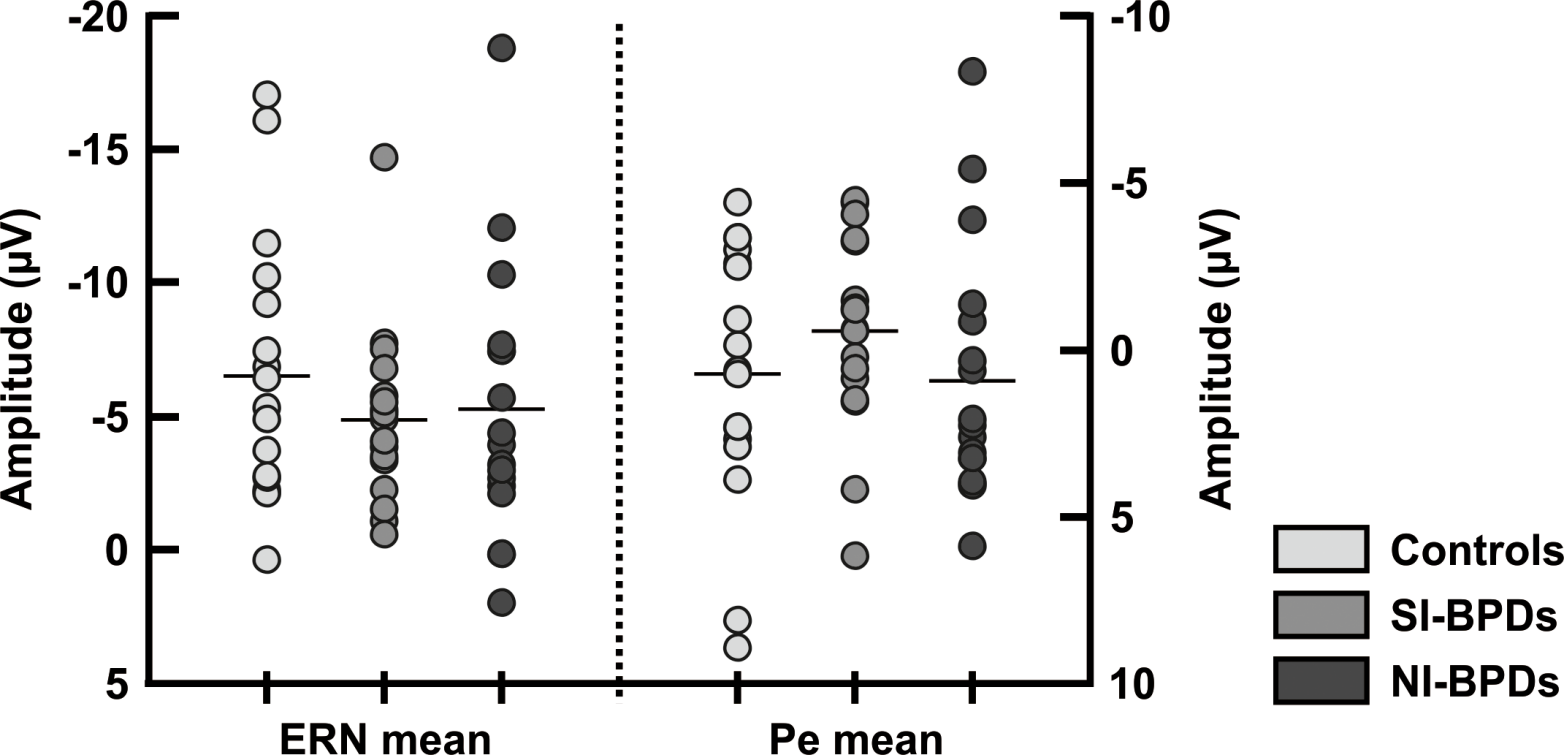
Mean amplitude distribution of the ERN and Pe for each participant, divided into the three groups of study (Controls, SI-BPD, NI-BPD). A clear overlapping of distributions can be seen.

Errors led to an increased negativity peaking about 50 ms after the error (see Figs 1 and 2), which was identified as the ERN component, with a clear fronto-central scalp distribution in all groups [18,22]. A repeated measures ANOVA (rmANOVA) including Group (Control, SI-BPD and NI-BPD) as a between-subject factor and Response (Correct *vs*. Error) and central Electrodes (Fz, Cz, and Pz) as within-subjects factors (mean amplitude measured at the time-window 30–80 ms) was performed. The increased negativity after errors, that characterizes the ERN component, was confirmed by the significant main effect of the Response [*F*(1,48) *=* 43.15, *p* < .001, *Ƞ* ^2^ = .473]. However, unexpectedly considering previous findings in the literature, no significant group differences were observed [Group: *F*(2,48) *=* .364, *p* = .697, *Ƞ* ^2^ = .015; Response x Group: *F*(2,48) *=* .416, *p* = .662, *Ƞ* ^2^ = .017]. Fig 3 shows the distribution of the ERN values for all the subjects of the three groups. As can be seen, the distribution in the three groups is very similar.

The Pe ERP component peaked around 200 ms (Figs 2 and 3).We conducted the same rmANOVA analysis as for the ERN using the mean amplitude measured at the time-window 185–265 milliseconds. The Pe was associated with errors trials as shown by a Response main effect [*F*(1,48) *=* 81.9, *p* < .001, *Ƞ* ^2^ = .631]. Visual inspection suggested a reduction of the Pe in SI-BPD group compared to the Control group and the NI-BPD group. However, no significant main effect of Group [*F*(2,48) *=* .9, *p* = .41, *Ƞ* ^2^ = .036] nor interaction Response x Group [*F*(2,48) *=* .48, *p* = .622, *Ƞ* ^2^ = .02] were found, showing no differences between groups in this ERP component. As can be observed in Figs 1 and 2, the amplitude of the Pe component considering the previous ERN peak seems to be reduced in BPDs groups, especially in the SI-BPD group. Nevertheless, we calculated the difference in amplitude between the ERN and the Pe peaks in the error trials for all subjects at Cz electrode, and discarded a reduced ERN-Pe amplitude for BPDs by means of an ANOVA analysis with Group as single factor [*F*(2,48) *=* 1.009, *p* = .372, *Ƞ* ^2^ = .04]. Fig 3 also shows the distribution of Pe amplitudes for the three groups.

In order to discard the effect of excess eye movement activity which could contaminate the results, we applied an rmANOVA in the EOG electrode. Ocular electrodes showed no significant differences between conditions in the ERN time range [*F*(1,48) = .160, *p* = .691, *Ƞ* ^2^ = .003], but significant differences between conditions in the Pe time range [*F*(1,48) = 7.742, *p* = .008, *Ƞ* ^2^ = .139]. However, neither the ERN [Group *F*(2,48) *=* 1.354, *p* = .268, *Ƞ* ^2^ = .053; Response x Group *F*(2,48) *=* 1.889, *p* = .162, *Ƞ* ^2^ = .073] nor the Pe time range [Group *F*(2,48) *=* .513, *p* = .602, *Ƞ* ^2^ = .021; Response x Group *F*(2,48) *=* .269, *p* = .765, *Ƞ* ^2^ = .011] showed significant effect of group, ruling out the possibility of a differential effect of eye movements in the data.

**Fig 4:**
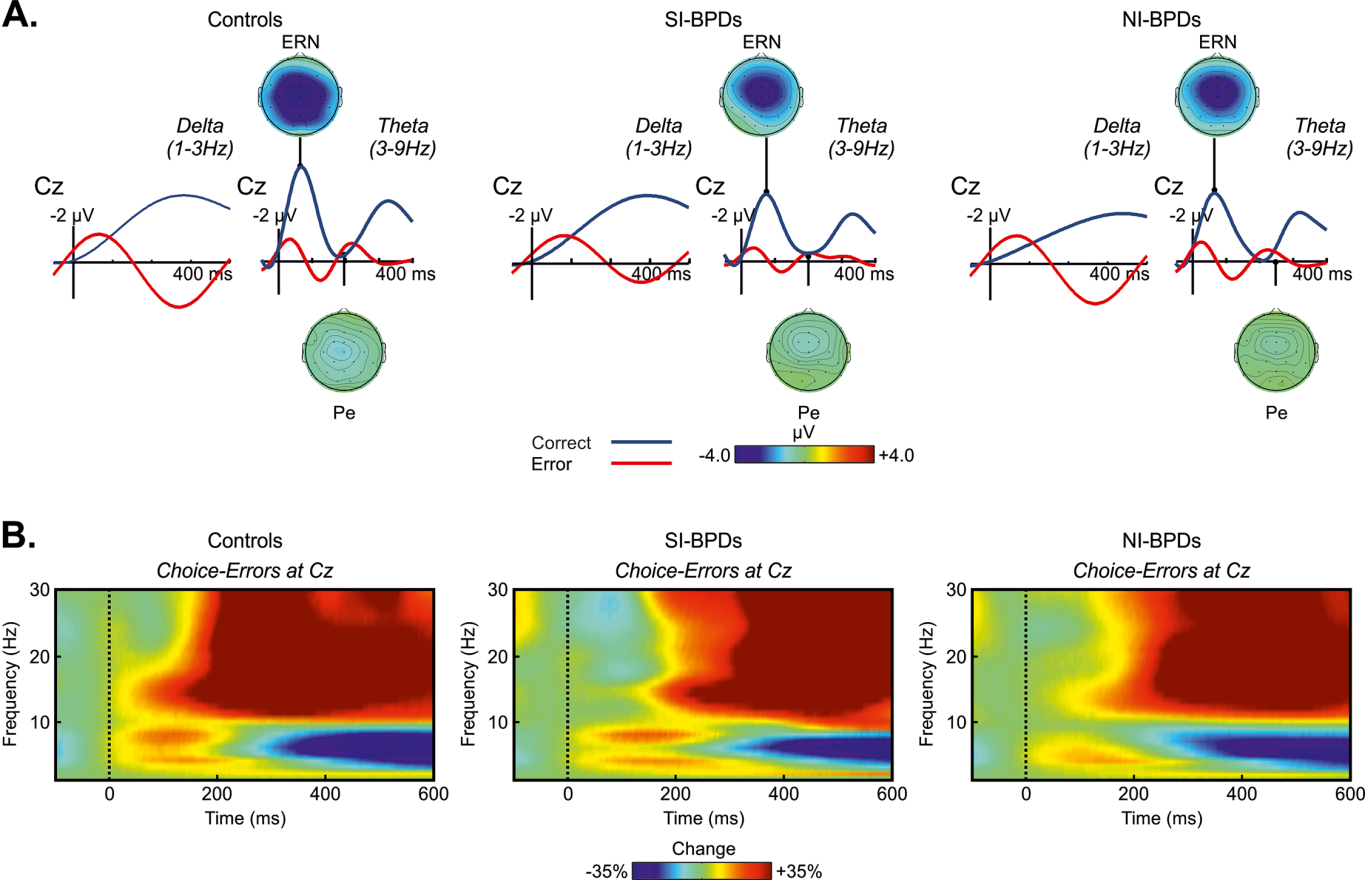
(A) Grand average of response-locked ERPs at Cz electrode, filtered for delta activity (3hz low pass), and for theta activity (3-9hz band pass), for controls, SI-BPD and NI-BPD individuals. Correct trials are depicted in blue solid lines and error trials in red lines. Scalp distribution for theta (3–9 Hz band pass filter) error activity were calculated for the two time windows (ERN and Pe), maximum and minimum values in microvolts are -4.0 and +4.0. (B) Grand average of spectral power modulation for the error trials at Cz electrode.

Finally, in order to discard the possibility of significant differences existing between groups in the ERPs of different frequency domains [56] we repeated the same analysis filtering the data to delta (1–3 Hz) and theta (3–9 Hz) frequency bands (Fig 4A). The rmANOVA revealed neither significant differences between groups [(SI-BPD, NI-BPD, Control) and (BPD, Controls)] nor congruently with non-filtered results (see supplementary results for details in S1 File).

### Time-Frequency: response-locked data

In order to study the effects in the power of theta band associated with error execution (see Fig 4B), a rmANOVA including Group (Control, SI-BPD, NI-BPD) as a between-subject factor and Response (correct vs. error responses) and central Electrodes (Fz, Cz, and Pz) as within-subjects factors (mean amplitude measured at the time-window 50–250 ms) was performed. The significant main effect of Response [*F*(1,42) = 178.7, *p* < .001, *Ƞ* ^2^ = .788] confirmed larger theta power for the error trials compared to the correct trials. As in the ERP analyses, no group differences were found between groups [Group: *F*(2,42) = .4, *p* = .672, *Ƞ* ^2^ = .016; Response x Group: *F*(2,42) = 1.948, *p* = .154, *Ƞ* ^2^ = .075].

### Medication Load

No differences were found in the total number of drugs used between BPD groups [SI- BPD group (2.59 ± 1.66); NI-BPD (2.27 ± 1.98); *t*(132) = .5, *p* = .621]. For a more detailed description of the medication see Supplementary Material (Table A in S1 File).

The medication load effects were tested both for ERPs and for the theta band power of Time-Frequency. First, the correlation analysis revealed no relationship between Medication Load and ERN [NI-BPD group (*r* = .122, *p* = .642); SI-BPD group (*r* = -.185, *p* = .476)] nor the Pe [NI-BPD group (*r* = .193; *p* = .457); SI-BPD group (*r* = -0.308, *p =* .229)] components. Second, no relationship was found between the Medication Load and the theta band power of Time-Frequency [NI-BPD group (*r* = .029, *p* = .913); SI-BPD (*r* = .048, *p* = .855)].

## Discussion

In the present manuscript we studied whether a large sample (N = 34) of well characterized BPD patients presented an executive dysfunction in error monitoring and if this problem could be associated with non-suicidal self-injury (NSSI) behaviors. The results showed very clearly that neural signatures of error processing (ERN, Pe and theta oscillatory activity) were not altered in BPD patients compared to healthy controls. In addition, no significant differences in behavioral measures of error rates, reaction time and corrective actions after the execution of an error were found. These results are contrary to our formulated hypothesis based on previous findings [27,28] and suggest preserved error monitoring mechanisms in BPD patients, independently of their NSSI behaviors.

Present findings contradict previous evidences which showed alteration of error monitoring in these patients when compared with healthy controls, especially a reduction in the ERN component amplitude [27,28]. Moreover, in contrast to a previous report by Ruchsow et al. [28], we did not find alterations in the Pe component or reaction times in relation to control participants. Additionally, although patients self-reported higher impulsivity than control participants, the behavioral performance on the Flanker task was similar between patients and controls and no significant differences were found in the inhibitory measures related to the stop signal (SSRT and post error slowing differences). These differences regarding the two previous studies [27,28] might be explained by the higher number of participants included in the present one (34 BPD *vs* 12 BPD patients in the two previous studies). In the same vein, the lack of alteration in error monitoring in BPD patients obtained here, is in convergence with previous inconsistent findings concerning executive functions in BPD [57]. These last results suggest that BPD executive functions are preserved in all sub-domains, except in working memory [58]. Complementarily, Hagenhoff et al. [59] did not find impairment in response inhibition nor error rates in BPD patients, which is also evidenced in the present paper. Thus, as proposed by others [58,60], response inhibition deficits might not be a core aspect in BPD at least considering standard laboratory measures as for example, the stop-signal task.

A novel approach of this study was the inclusion of two groups of BPD patients, one with NSSI history and another without it. Despite BPD patients who engage in NSSI behaviors showing high clinical severity and functional impairment (in comparison with NI-BPD group, as shown in DIB-R, CGI-BPD and GAF scores), behavioral measures (except reaction time for erroneous responses), ERN and Pe amplitudes and theta power increase were similar in these two groups of patients. Thus, beyond the possible impact of NSSI behaviors in everyday life, BPD patients who self-harm, have preserved error monitoring mechanisms when compared with healthy controls and BPD patients without history of NSSI behaviors. In this same line, Janis and Nock [61] reported no differences in performance-based measures of impulsiveness in NSSI individuals, showing that they are, perhaps, impulsive only in certain situations. Indeed, BPD patients have shown alterations in their fronto-limbic neural activity patterns, during the performance of behavioral tasks under negative emotional induction (*e*.*g*. verbal salient stimuli in a go/no-go task, [62]; performing a go/no-go task after anger induction [63]).

Therefore, given this finding, NSSI behaviors could not be explained by a dysfunction in error monitoring. This is congruent with the idea that these behaviors respond to a variety of functions and, importantly, that not all self-injurers engaged in this behavior act impulsively and “out of control” (that is, associated with a lack of executive control: [64,65]), but they might spend some time thinking about NSSI before engaging in it as an emotional self-regulation strategy [4,8,31]. Consequently, to understand why these complex behaviors are maintained (which is very interesting because they are not an isolated act), it is important to consider that BPD patients would incur in NSSI behaviors not as a consequence of a systematic failure in the internal error signals processing (ERN, Pe), but because their contingencies are reinforced (*e*.*g*. feel alive, stop arguing, [8]). This hypothesis is congruent with Linehan’s biosocial theory [66], insomuch as the NSSI behaviors are maladaptive attempts to self-regulate negative emotional states which, in turn, are positively and/or negatively reinforced by their outcomes. It is important to notice that because of their preserved error monitoring system, the learning of alternative self-regulating strategies (more adaptive than NSSI) is possible in most BPD patients who undergo a psychological treatment [10,67], showing that they are able to process the internal error signals adequately, in contrast to the external feedbacks [68–70].

The main limitation of the present study arises from the fact that BPD patients included were undergoing psychopharmacological treatment. Despite being ecologically valid, it is known that psychopharmacological compounds could play a confounding effect on the ERN [71,72]. Importantly, we used a medication load scale which showed no relationship between behavioral and electrophysiological measures. Another potential limitation of the present data arises from uncontrolled co-morbidities, more especially ADHD which were related with deficits in executive functions [60,73]. Finally, all participants were females and, in consequence, the present results cannot be generalized to males due to the gender differences in executive functioning [74] and in the ERN component [75].

In summary, present results show that error monitoring mechanisms are not a core aspect of BPD or NSSI behaviors. Therefore, in an attempt to self-regulate, the NSSI are not impulsive behaviors associated with the failure of a primary mechanism in performance monitoring, but with more complex interactions (*e*.*g*. information processing distortion, long lasting traits, emotional avoidance patterns). These results are encouraging because they show that BPD patients are able to detect, monitor and inhibit these behaviors. They also allow a better understanding of these complex and disabling behaviors, which are a public health concern and pose a therapeutic challenge.

## Supporting Information

S1 File Relevant data which complement the findings described in manuscript. Medication prescription of BPD patients (Table A). Comorbidity in the BPD group (Tabe B). Behavioral data (Figure A).
(DOCX)

## References

[1] Lenzenweger MF, Lane MC, Loranger AW, Kessler RC (2007) DSM-IV personality disorders in the National Comorbidity Survey Replication. Biol Psychiatry 62: 553–564. 17217923 10.1016/j.biopsych.2006.09.019 PMC2044500

[2] Zanarini MC, Frankenburg FR, Reich DB, Fitzmaurice G, Weinberg I, Gunderson JG (2008) The 10-year course of physically self-destructive acts reported by borderline patients and axis II comparison subjects. Acta Psychiatr Scand 117: 177–184. 10.1111/j.1600-0447.2008.01155.x 18241308 PMC3884820

[3] Nock MK, Prinstein MJ (2004) A functional approach to the assessment of self-mutilative behavior. J Consult Clin Psychol 72: 885–890. 15482046 10.1037/0022-006X.72.5.885

[4] Klonsky ED (2007) The functions of deliberate self-injury: a review of the evidence. Clin Psychol Rev 27: 226–239. 17014942 10.1016/j.cpr.2006.08.002

[5] Klonsky ED (2011) Non-suicidal self-injury in United States adults: prevalence, sociodemographics, topography and functions. Psychol Med 41: 1981–1986. 10.1017/S0033291710002497 21208494

[6] American Psychiatric Association (2013) Diagnostic and Statistical Manual of Mental Disorders (5th ed.). Arlington, VA: American Psychiatric Publishing.

[7] American Psychiatric Association (2000) Diagnostic and Statistical Manual of Mental Disorders (revised 4th ed.). Washington, DC: American Psychiatric Association.

[8] Nock MK (2010) Self-Injury. Annu Rev Clin Psychol 6: 339–363. 10.1146/annurev.clinpsy.121208.131258 20192787

[9] Linehan MM, Heard HL, Armstrong HE (1993) Naturalistic follow-up of a behavioral treatment for chronically parasuicidal borderline patients. Arch Gen Psychiatry 50: 971–974. 8250683 10.1001/archpsyc.1993.01820240055007

[10] Linehan MM (1987) Dialectical Behavioral Therapy: A Cognitive Behavioral Approach to Parasuicide. J Pers Disord 1: 328–333.

[11] Zanarini MC, Laudate CS, Frankenburg FR, Wedig MM, Fitzmaurice G (2013) Reasons for Self-Mutilation Reported by Borderline Patients Over 16 Years of Prospective Follow-Up. J Pers Disord 27: 1–12. 23795756 10.1521/pedi_2013_27_115 PMC3876880

[12] Glenn CR, Klonsky ED (2009) Emotion dysregulation as a core feature of borderline personality disorder. J Pers Disord 23: 20–28. 10.1521/pedi.2009.23.1.20 19267659

[13] Carpenter RW, Trull TJ (2013) Components of emotion dysregulation in borderline personality disorder: a review. Curr Psychiatry Rep 15: 335 10.1007/s11920-012-0335-2 23250816 PMC3973423

[14] Mak ADP, Lam LCW (2013) Neurocognitive profiles of people with borderline personality disorder. Current Opinion in Psychiatry 26: 90–96. 10.1097/YCO.0b013e32835b57a9 23196999

[15] Fikke LT, Melinder A, Landrø NI (2011) Executive functions are impaired in adolescents engaging in non-suicidal self-injury. Psychol Med 41: 601–610. 10.1017/S0033291710001030 20482935

[16] Ullsperger M (2006) Performance monitoring in neurological and psychiatric patients. Int J Psychophysiol 59: 59–69. 16288812 10.1016/j.ijpsycho.2005.06.010

[17] Falkenstein M, Hohnsbein J, Hoormann J, Blanke L (1990) Effects of errors in choice reaction tasks on the ERP under focused and divided attention In: Brunia C, Gaillard A, Kok A, editors. Psychophysiological Brain Research. Tilburg: Tilburg University Press, pp. 192–195.

[18] Gehring WJ, Goss B, Coles MGH, Meyer DE, Donchin E (1993) A Neural System for Error Detection and Compensation. Psychol Sci 4: 385–390.

[19] Holroyd CB, Dien J, Coles MG (1998) Error-related scalp potentials elicited by hand and foot movements: evidence for an output-independent error-processing system in humans. Neurosci Lett 242: 65–68. 9533395 10.1016/s0304-3940(98)00035-4

[20] Yeung N, Botvinick MM, Cohen JD (2004) The neural basis of error detection: conflict monitoring and the error-related negativity. Psychol Rev 111: 931–959. 15482068 10.1037/0033-295x.111.4.939

[21] Holroyd CB, Coles MGH (2002) The neural basis of human error processing: reinforcement learning, dopamine, and the error-related negativity. Psychol Rev 109: 679–709. 12374324 10.1037/0033-295X.109.4.679

[22] Falkenstein M, Hohnsbein J, Hoormann J, Blanke L (1991) Effects of crossmodal divided attention on late ERP components. II. Error processing in choice reaction tasks. Electroencephalogr Clin Neurophysiol 78: 447–455. 1712280 10.1016/0013-4694(91)90062-9

[23] Falkenstein M, Hoormann J, Christ S, Hohnsbein J (2000) ERP components on reaction errors and their functional significance: a tutorial. Biol Psychol 51: 87–107. 10686361 10.1016/s0301-0511(99)00031-9

[24] Nieuwenhuis S, Ridderinkhof KR, Blom J, Band GP, Kok A (2001) Error-related brain potentials are differentially related to awareness of response errors: evidence from an antisaccade task. Psychophysiology 38: 752–760. 11577898

[25] Manoach DS, Agam Y (2013) Neural markers of errors as endophenotypes in neuropsychiatric disorders. Front Hum Neurosci 7: e350. 10.3389/fnhum.2013.00350 PMC3714549 23882201

[26] Olvet DM, Hajcak G (2008) The error-related negativity (ERN) and psychopathology: Toward an Endophenotype. Clin Psychol Rev 28: 1343–1354. 10.1016/j.cpr.2008.07.003 18694617 PMC2615243

[27] De Bruijn ERA, Grootens K, Verkes RJ, Buchholz V, Hummelen J, Hulstijn W (2006) Neural correlates of impulsive responding in borderline personality disorder: ERP evidence for reduced action monitoring. J Psychiatr Res 40: 428–437. 16257009 10.1016/j.jpsychires.2005.09.004

[28] Ruchsow M, Walter H, Buchheim A, Martius P, Spitzer M, Kiefer M, et al (2006) Electrophysiological correlates of error processing in borderline personality disorder. Biol Psychol 72: 133–140. 16288950 10.1016/j.biopsycho.2005.08.006

[29] Jollant F, Bellivier F, Leboyer M, Astruc B, Castelnau D, Verdier R, et al (2005) Impaired Decision Making in Suicide Attempters. Am J Psych 162: 304–310. 10.1176/appi.ajp.162.2.304 15677595

[30] Dougherty DM, Mathias CW, Marsh-Richard DM, Prevette KN, Dawes MA, Hatzis ES, et al (2009) Impulsivity and clinical symptoms among adolescents with non-suicidal self-injury with or without attempted suicide. Psychiatry Res 169: 22–27. 10.1016/j.psychres.2008.06.011 19631392 PMC3062197

[31] Chapman AL, Gratz KL, Brown MZ (2006) Solving the puzzle of deliberate self-harm: the experiential avoidance model. Behav Res Ther 44: 371–394. 16446150 10.1016/j.brat.2005.03.005

[32] Zanarini MC, Frankenburg FR, Reich DB, Fitzmaurice G, Weinberg I, Gunderson JG (2008) The 10-year course of physically self-destructive acts reported by borderline patients and axis II comparison subjects. Acta Psychiatr Scand 117: 177–184. 10.1111/j.1600-0447.2008.01155.x 18241308 PMC3884820

[33] Barrachina J, Soler J, Campins MJ, Tejero A, Pascual JC, Alvarez E, et al (2004) Validation of a Spanish version of the Diagnostic Interview for Bordelines-Revised (DIB-R). Actas Españolas Psiquiatr 32: 293–298. 15529214

[34] Pérez-Prieto F, Alvarez I, Monros P, Sarria C, Pérez-Marín E, et al. (2008) Adaptación española de la SCID-II. Valencia.

[35] First MB, Gibbon M (1997) User’s Guide for the Structured Clinical Interview for DSM-IV Axis I Disorders SCID-I: Clinician Version. Washington, DC: American Psychiatric Press.

[36] Klonsky ED, Glenn CR (2008) Assessing the Functions of Non-suicidal Self-injury: Psychometric Properties of the Inventory of Statements About Self-injury (ISAS). J Psychopathol Behav Assess 31: 215–219. 29269992 10.1007/s10862-008-9107-z PMC5736316

[37] Glenn CR, Klonsky ED (2011) One-year test-retest reliability of the Inventory of Statements about Self-Injury (ISAS). Assessment 18: 375–378. 10.1177/1073191111411669 21665881

[38] Bohus M, Kleindienst N, Limberger MF, Stieglitz R-D, Domsalla M, Chapman AL, et al (2009) The short version of the Borderline Symptom List (BSL-23): development and initial data on psychometric properties. Psychopathology 42: 32–39. 10.1159/000173701 19023232

[39] Soler J, Vega D, Feliu-Soler A, Trujols J, Soto A, Elices M, et al (2013) Validation of the Spanish version of the Borderline Symptom List, short form (BSL-23). BMC Psychiatry 13: e139. 10.1186/1471-244X-13-139 PMC3658905 23672691

[40] Perez V, Barrachina J, Soler J, Pascual JC, Campins MJ, Puigdemont D, et al (2007) The clinical global impression scale for borderline personality disorder patients (CGI-BPD): a scale sensible to detect changes. Actas Españolas Psiquiatr 35: 229–235. 17592784

[41] Patton JH, Stanford MS, Barratt ES (1995) Factor structure of the Barratt impulsiveness scale. J Clin Psychol 51: 768–774. 8778124 10.1002/1097-4679(199511)51:6<768::aid-jclp2270510607>3.0.co;2-1

[42] Vederman AC, Weisenbach SL, Rapport LJ, Leon HM, Haase BD, Franti LM, et al (2012) Modality-specific alterations in the perception of emotional stimuli in Bipolar Disorder compared to Healthy Controls and Major Depressive Disorder. Cortex 48: 1027–1034. 10.1016/j.cortex.2011.03.017 21683948 PMC3660134

[43] Sackeim HA (2001) The definition and meaning of treatment-resistant depression. J Clin Psychiatry 62: 10–17. 11480879

[44] Almeida JRC, Akkal D, Hassel S, Travis MJ, Banihashemi L, Kerr N, et al (2009) Reduced gray matter volume in ventral prefrontal cortex but not amygdala in bipolar disorder: significant effects of gender and trait anxiety. Psychiatry Res 171: 54–68. 10.1016/j.pscychresns.2008.02.001 19101126 PMC2646161

[45] Davis JM, Chen N (2004) Dose Response and Dose Equivalence of Antipsychotics. J Clin Psychopharmacol 24: 192–208. 15206667 10.1097/01.jcp.0000117422.05703.ae

[46] Eriksen BA, Eriksen CW (1974) Effects of noise letters upon the identification of a target letter in a nonsearch task. Percept Psychophys 16: 143–149.

[47] Rodriguez-Fornells A, Kurzbuch AR, Münte TF (2002) Time course of error detection and correction in humans: neurophysiological evidence. J Neurosci 22: 9990–9996. 12427856 10.1523/JNEUROSCI.22-22-09990.2002 PMC6757828

[48] Krämer UM, Cunillera T, Càmara E, Marco-Pallarés J, Cucurell D, Nager W, et al (2007) The impact of catechol-O-methyltransferase and dopamine D4 receptor genotypes on neurophysiological markers of performance monitoring. J Neurosci 27: 14190–14198. 18094258 10.1523/JNEUROSCI.4229-07.2007 PMC6673506

[49] Band GPH, van der Molen MW, Logan GD (2003) Horse-race model simulations of the stop-signal procedure. Acta Psychol 112: 105–142. 50. 10.1016/s0001-6918(02)00079-3 12521663

[50] Band GP, van Boxtel GJ (1999) Inhibitory motor control in stop paradigms: review and reinterpretation of neural mechanisms. Acta Psychol 101: 179–211. 10.1016/s0001-6918(99)00005-0 10344185

[51] Joyce CA, Gorodnitsky IF, Kutas M (2004) Automatic removal of eye movement and blink artifacts from EEG data using blind component separation. Psychophysiology 4: 313–325. 10.1111/j.1469-8986.2003.00141.x 15032997

[52] Krämer UM, Cunillera T, Càmara E, Marco-Pallarés J, Cucurell D, Nager W, et al (2007) The impact of catechol-O-methyltransferase and dopamine D4 receptor genotypes on neurophysiological markers of performance monitoring. J Neurosci 27:14190–14198. 18094258 10.1523/JNEUROSCI.4229-07.2007 PMC6673506

[53] Vilà-Balló A, Hdez-Lafuente P, Rostan C, Cunillera T, Rodriguez-Fornells A (2014) Neurophysiological correlates of error monitoring and inhibitory processing in juvenile violent offenders. Biol Psychol 102:141–152. 10.1016/j.biopsycho.2014.07.021 25108171

[54] Jennings RJ, Wood CC (1976) The ɛ-Adjustment Procedure for Repeated-Measures Analyses of Variance. Psychophysiology 13: 277–278. 1273235 10.1111/j.1469-8986.1976.tb00116.x

[55] Tallon-Baudry C, Bertrand O, Delpuech C, Permier J (1997) Oscillatory gamma-band (30–70 Hz) activity induced by a visual search task in humans. J Neurosci 17: 722–734. 8987794 10.1523/JNEUROSCI.17-02-00722.1997 PMC6573221

[56] Bernat EM, Nelson LD, Steele VR, Gehring WJ, Patrick CJ (2011) Externalizing psychopathology and gain-loss feedback in a simulated gambling task: dissociable components of brain response revealed by time-frequency analysis. J Abnorm Psychol 120: 352–364. 10.1037/a0022124 21319875 PMC3092030

[57] LeGris J, Links PS, van Reekum R, Tannock R, Toplak M (2012) Executive function and suicidal risk in women with Borderline Personality Disorder. Psychiatry Res 196: 101–108. 10.1016/j.psychres.2011.10.008 22377570

[58] Lampe K, Konrad K, Kroener S, Fast K, Kunert HJ, Herpertz SC, et al (2007) Neuropsychological and behavioural disinhibition in adult ADHD compared to borderline personality disorder. Psychol Med 37: 1717–1729. 17506923 10.1017/S0033291707000517

[59] Hagenhoff M, Franzen N, Koppe G, Baer N, Scheibel N, Sammer G, et al (2013) Executive functions in borderline personality disorder. Psychiatry Res 210: 1–8. 23764434 10.1016/j.psychres.2013.05.016

[60] Krause-Utz A, Sobanski E, Alm B, Valerius G, Kleindienst N, Bohus M, et al (2013) Impulsivity in relation to stress in patients with borderline personality disorder with and without co-occurring attention-deficit/hyperactivity disorder: an exploratory study. J Nerv Ment Dis 201: 116–123. 10.1097/NMD.0b013e31827f6462 23364120

[61] Janis IB, Nock MK (2009) Are self-injurers impulsive?: Results from two behavioral laboratory studies. Psychiatry Res 169: 261–267. 10.1016/j.psychres.2008.06.041 19758706 PMC2766846

[62] Silbersweig D, Clarkin JF, Goldstein M, Kernberg OF, Tuescher O, Levy KM, et al (2007) Failure of Frontolimbic Inhibitory Function in the Context of Negative Emotion in Borderline Personality Disorder. Am J Psychiatry 164: 1832–1841. 18056238 10.1176/appi.ajp.2007.06010126

[63] Holtmann J, Herbort MC, Wüstenberg T, Soch J, Richter S, Walter H, et al (2013) Trait anxiety modulates fronto-limbic processing of emotional interference in borderline personality disorder. Front Hum Neurosci 7: e54. 10.3389/fnhum.2013.00054 PMC3585713 23459637

[64] Herpertz S (1995) Self-injurious behaviour. Psychopathological and nosological characteristics in subtypes of self-injurers. Acta Psychiatr Scand 91: 57–68. 7754789 10.1111/j.1600-0447.1995.tb09743.x

[65] Herpertz S, Sass H, Favazza A (1997) Impulsivity in self-mutilative behavior: psychometric and biological findings. J Psychiatr Res 31: 451–465. 9352472 10.1016/s0022-3956(97)00004-6

[66] Crowell SE, Beauchaine TP, Linehan MM (2009) A biosocial developmental model of borderline personality: Elaborating and extending Linehan’s theory. Psychol Bull 135: 495–510. 10.1037/a0015616 19379027 PMC2696274

[67] Linehan MM, Armstrong HE, Suarez A, Allmon D, Heard HL (1991) Cognitive-Behavioral Treatment of Chronically Parasuicidal Borderline Patients. Arch Gen Psychiatry 48: 1060–1064. 1845222 10.1001/archpsyc.1991.01810360024003

[68] King-Casas B, Sharp C, Lomax-Bream L, Lohrenz T, Fonagy P, Montague PR (2008) The rupture and repair of cooperation in borderline personality disorder. Science 321: 806–810. 10.1126/science.1156902 18687957 PMC4105006

[69] Schuermann B, Kathmann N, Stiglmayr C, Renneberg B, Endrass T (2011) Impaired decision making and feedback evaluation in borderline personality disorder. Psychol Med 41: 1917–1927. 10.1017/S003329171000262X 21262034

[70] Vega D, Soto A, Amengual JL, Ribas J, Torrubia R, Rodríguez-Fornells A, et al (2013) Negative reward expectations in Borderline Personality Disorder patients: Neurophysiological evidence. Biol Psychol 94: 388–396. 10.1016/j.biopsycho.2013.08.002 23969232

[71] De Bruijn ERA, Hulstijn W, Verkes RJ, Ruigt GSF, Sabbe BGC (2004) Drug-induced stimulation and suppression of action monitoring in healthy volunteers. Psychopharmacology 177: 151–160. 15578258 10.1007/s00213-004-1915-6

[72] De Bruijn ERA, Sabbe BGC, Hulstijn W, Ruigt GSF, Verkes RJ (2006) Effects of antipsychotic and antidepressant drugs on action monitoring in healthy volunteers. Brain Res 1105: 122–129. 16499887 10.1016/j.brainres.2006.01.006

[73] Willcutt EG, Doyle AE, Nigg JT, Faraone SV, Pennington BF (2005) Validity of the executive function theory of attention-deficit/hyperactivity disorder: a meta-analytic review. Biol Psychiatry 57: 1336–1346. 15950006 10.1016/j.biopsych.2005.02.006

[74] Bolla KI, Eldreth DA, Matochik JA, Cadet JL (2004) Sex-related differences in a gambling task and its neurological correlates. Cereb Cortex 14: 1226–1232. 15142963 10.1093/cercor/bhh083

[75] Moran TP, Taylor D, Moser JS (2012) Sex moderates the relationship between worry and performance monitoring brain activity in undergraduates. Int J Psychophysiol 85: 188–194. 10.1016/j.ijpsycho.2012.05.005 22659221

