# Supplementary material for: Preserved Error-Monitoring in Borderline Personality Disorder Patients with and without Non-Suicidal Self-Injury Behaviors
Source: PLoS One. 2015 Dec 4;10(12):e0143994. doi: 10.1371/journal.pone.0143994 (PMC4670111; doi:10.1371/journal.pone.0143994)
Supplement: S1 File — Medication prescription of BPD patients (Table A). Comorbidity in the BPD group (Tabe B). Behavioral data (Figure A). (DOCX) [file pone.0143994.s001.docx]

**Preserved Error-Monitoring in Borderline Personality Disorder Patients with and without Non-Suicidal Self-Injury Behaviors**

Daniel Vega, AdriàVilà-Balló, Àngel Soto, Julià Amengual, Joan Ribas, Rafael Torrubia, Antoni Rodríguez-Fornells and Josep Marco-Pallarés

***- Supporting Information -***

**Table A.** Medication prescription of BPD patients

|  | NI-BPD  (n = 17) | |  | SI-BPD  (n = 17) | |  | Group differences | |
| --- | --- | --- | --- | --- | --- | --- | --- | --- |
|  | N | % |  | N | % |  | *Chi^2^* | *p* |
| Medication |  |  |  |  |  |  |  |  |
| SSRI | 8 | 47.1 |  | 8 | 47.1 |  | 0 | 1 |
| ATP | 5 | 29.4 |  | 5 | 29.4 |  | 0 | 1 |
| BZD | 8 | 47.1 |  | 11 | 64.7 |  | 1.07 | .300 |
| MS | 8 | 47.1 |  | 9 | 52.9 |  | .12 | .731 |

Notes. *SSRI*, Selective serotonin reuptake inhibitors; *ATP*, antipsychotic medications; *BZD*, benzodiazepines; *MS*, mood-stabilizing drugs.

**Table B.** Comorbidity in the BPD group

|  | NI-BPD  (n = 17) | |  | SI-BPD  (n = 17) | |
| --- | --- | --- | --- | --- | --- |
|  | N | % |  | N | % |
| **DSM-IV Axis I** |  |  |  |  |  |
| Past |  |  |  |  |  |
| MDD | 7 | 41.2 |  | 9 | 52.9 |
| Any anxiety disorder^a^ | 2 | 11.8 |  | 3 | 17.6 |
| Eating disorder^b^ | 4 | 23.5 |  | 3 | 17.6 |
| Substance abuse^c^ | 4 | 23.5 |  | 6 | 35.3 |
| Current |  |  |  |  |  |
| Any anxiety disorder^a^ | 2 | 11.8 |  | 7 | 41.2 |
| Eating disorder^b^ | 4 | 23.5 |  | 4 | 23.5 |
| Substance abuse^c^ | 4 | 23.5 |  | 8 | 47.1 |
| Other^d^ | 2 | 11.8 |  | 2 | 11.8 |
| **DSM-IV Axis II** |  |  |  |  |  |
| Cluster A^e^ | 0 | 0 |  | 5 | 29.4 |
| Paranoid | 0 | 0 |  | 4 | 23.5 |
| Schizotypal | 0 | 0 |  | 2 | 11.8 |
| Cluster B^f^ | 1 | 5.9 |  | 6 | 35.3 |
| Histrionic | 0 | 0 |  | 1 | 5.9 |
| Antisocial | 1 | 5.9 |  | 5 | 29.4 |
| Cluster C^g^ | 5 | 29.4 |  | 4 | 23.6 |
| Avoidant | 1 | 5.9 |  | 2 | 11.8 |
| Dependent | 5 | 29.4 |  | 2 | 11.8 |

Notes. *MDD*, Major depressive disorder.

^a^ Any anxiety disorder

^b^ Any eating disorder

^c^ Any substance use disorder

^d^ Any other disorder such as adaptive disorder

^e^ Any cluster A personality disorder (paranoid, schizoid or schizotypal)

^f^ Any cluster B personality disorder (antisocial, histrionic or narcissistic)

^g^ Any cluster C personality disorder (obsessive-compulsive, dependent or avoidant)

**Behavioral Results**

**
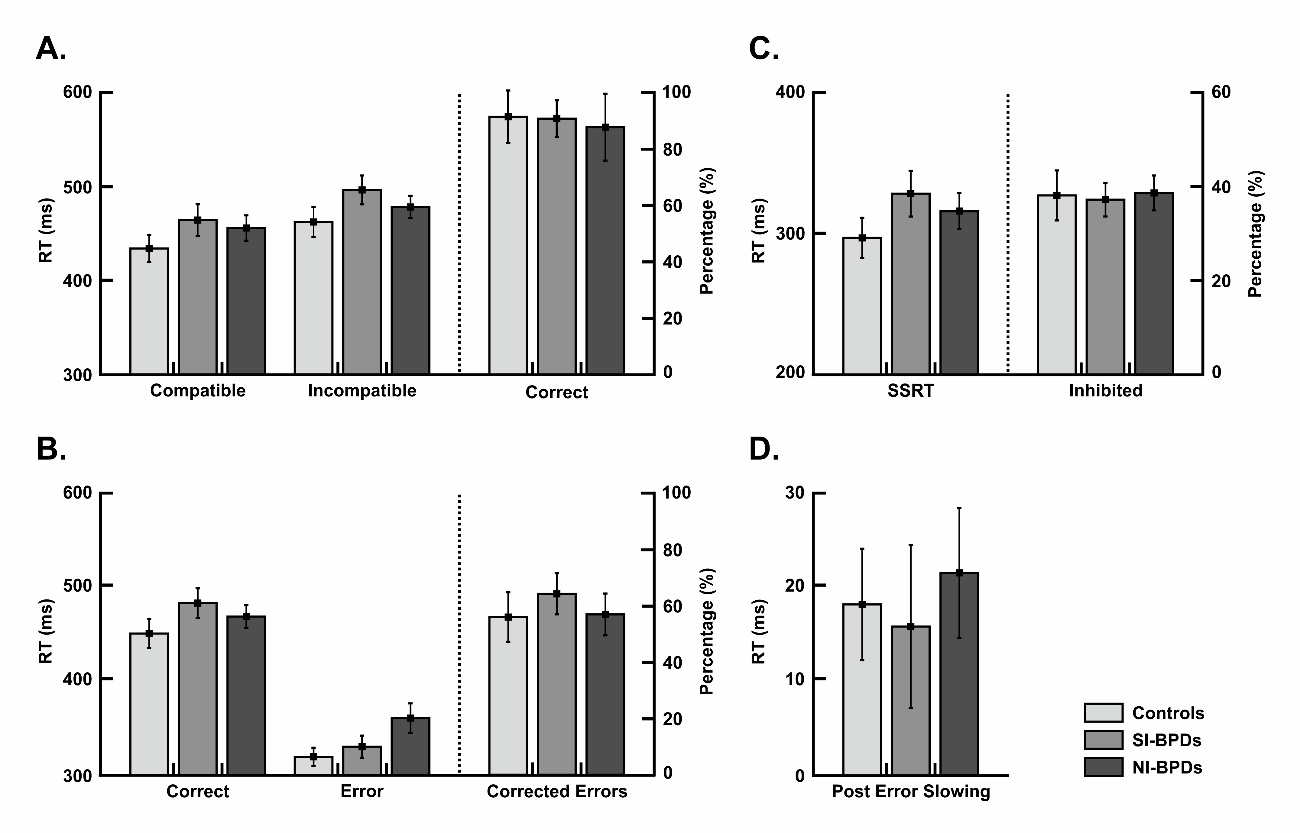
**

**Figure A. Behavioral data for controls (light grey), SI-BPD (grey) and NI-BPD (dark grey) individuals**. A. Mean reaction times (±SEM) from completed correct compatible and incompatible trials; and mean percentage (±SEM) of correct trials from go condition. B. Mean reaction times (±SEM) from completed correct and error trials; and mean percentage (±SEM) of corrected errors from go trials. C. Mean reaction times (±SEM) from Stop Signal; and mean percentage (±SEM) of inhibited from stop trials. D. Mean of Post Error Slowing reaction times (±SEM).

**Filtered Response-locked ERP data**

To discard differences in the response-locked activity between the Control and two BPD groups, we filtered the ERP response-locked data to differentiate between the activity associated to the delta (1-3 Hz) and theta frequencies (3-9 Hz).

In addition, we firstly entered the theta-ERN mean amplitude (measured between 30-80 ms) and theta-Pe mean amplitude (measured between 185-265ms) in the same rmANOVA as the not filtered analysis. The increase in the theta-ERN activity after errors was confirmed by the significant main effect of Response [*F*(1,48) *=* 71.844, *p* < .001, *Ƞ*^2^ = .599]. Importantly, no main effect of Group [*F*(2,48) *=* .852, *p* = .433, *Ƞ*^2^ = .034] nor interaction Response x Group [*F*(2,48) *=* .769, *p* = .469, *Ƞ*^2^ = .031] were found, showing no theta-ERN amplitude differences between groups. On the other hand, no differences between correct and error trials were found in the theta-Pe time-window [main effect of Response: *F*(1,48) *=* 2.53, *p* = .12, *Ƞ*^2^ = .05], as well as no significant main effect of Group [*F*(2,48) *=* 1.8, *p* = .18, *Ƞ*^2^ = .07] nor interaction Response x Group effect [*F*(2,48) *=* .861, *p* = .429, *Ƞ*^2^ = .035] were encountered.

Secondly, we entered the delta-ERN mean (measured between 30-80 ms) and delta-Pe (mean amplitude measured between 185-265 ms) amplitudes in the subsequent rmANOVA. No significant main effect of Response was found between the correct and error trials for delta-ERN [*F*(1,48) *=* 2.7, *p* = .11, *Ƞ*^2^ = .053], and no group differences were found as there were no main effect of Group [*F*(2,48) *=* .03, *p* = .971, *Ƞ*^2^ = .001] nor Response x Group interaction [*F*(2,48) *=* .024, *p* = .976, *Ƞ*^2^ = .001]. Error trials showed significant main effect of condition in the delta-Pe [*F*(1,48) *=* 75.889, *p* < .001; *Ƞ*^2^ = 0.613]. Importantly, no delta-Pe differences were found between groups [main effect of Group: *F*(2,48) *=* 1.025, *p* = .367, *Ƞ*^2^ = .041; interaction Response x Group: *F*(2,48) *=* .596, *p* = .555; , *Ƞ*^2^ = .024].
